# Study on the Microstructure of the New Paste of Recycled Aggregate Self-Compacting Concrete

**DOI:** 10.3390/ma13092114

**Published:** 2020-05-02

**Authors:** Kheira Zitouni, Assia Djerbi, Abdelkader Mebrouki

**Affiliations:** 1 Civil Engineering and Architecture Department-LCTPE, Faculty of Sciences and Technology, Abdelhamid Ibn-Badis University, 27000 Mostaganem, Algeria; kheira.zitouni@hotmail.com (K.Z.); mebroukiaek@yahoo.fr (A.M.); 2 MAST Department-FM2D, University Gustave Eiffel, IFSTTAR, F-77447 Marne la Vallée, France

**Keywords:** recycled aggregate, self-compacting concrete, mercury intrusion porosimetry, SEM observation, new paste, compressive strength

## Abstract

Previous literature indicates a decrease in the mechanical properties of various concrete types that contain recycled aggregates (RA), due to their porosity and to their interface of transition zone (ITZ). However, other components of the RA concrete microstructure have not yet been explored, such as the modification of the new paste (NP) with respect to a reference concrete. This paper deals with the microstructure of the new paste of self-compacting concrete (SCC) for different levels of RA. The water to binder ratio (w/b) was kept constant for all concrete mixtures, and equal to 0.5. The SCC mixtures were prepared with percentages of coarse RA of 0%, 30%, 50% and 100%. Mercury intrusion porosimetry test (MIP) and scanning electron microscope (SEM) observations were conducted on the new paste of each concrete. The results indicated that the porosity of the new paste presents a significant variation for replacement percentages of 50% and 100% with respect to NP0 and NP30. However, RA contributed to the refinement of the pore structure of the new paste. The amount of macrospores the diameter of which is in the 50–10,000 nm range was reduced to 20% for NP50 and NP100, while it was about 30% for NP0 and NP30, attributed to the water released by RA. Compressive strength loss for SCC50 and SCC100 concretes are both influenced by porosity of RA, and by the NP porosity. The latter is similar for these two concretes with the 26% increase compared to a reference concrete.

## 1. Introduction

In the context of sustainable development, recycling of concrete waste is carried out with the main purpose of protecting the environment through the reduction of generated greenhouse gases and the preservation of natural resources. The reuse of concrete from demolition as aggregates in new concrete compositions reduces overheads and eases costs associated to waste management. Thus, this approach promotes the protection of natural resources, which are becoming increasingly difficult to obtain. Recycling of demolition waste has then witnessed a rather significant development. The European Directive on waste (2008/98/EC) [1] has fixed thresholds of waste from deconstruction/demolition to be implemented in new concretes at 70% by 2020.

Using recycled aggregates (RA) in self-compacting concrete (SCC) improves the ecological impact of concrete, since their microstructure properties are greater than normal vibrated concrete [2,3,4]. The use of the fine content in SCC refines the microstructure and hence the pore network of the material, which improves SCC mixtures’ durability compared to that of ordinary vibrated concrete [2]. In fact, the replacement of natural aggregates with RA can affect the other properties of SCCs. Some research has been carried out for a constant volume of binder in order to evaluate the effect of the replacement rate of RA and to quantify their effects on different properties [5,6,7,8,9]. Sasanipour et al. [5] showed that the replacement of 25% of coarse aggregates had no significant effect on the durability properties of self-compacting concrete including electrical resistivity and chloride ion resistance. However, the use of silica fume improved the durability performance of SCC-containing recycled aggregates [6]. Pereira-de-Oliveira et al. [7] concluded it is viable to replace natural coarse aggregates by recycled coarse aggregates in SCCs. Their results showed that the recycled coarse aggregate incorporation did not significantly affect the water permeability, while water capillarity coefficient is slightly decreased when 100% of coarse RA is used. In addition, the water penetration depth is reduced when increasing the amount of RA in SCC. Kou et al. [8] found that the properties of the SCCs made from river sand and crushed recycled aggregates showed only slight differences with respect to a reference concrete. However, Omrane et al. [9] showed the RA content should be limited to 50% to achieve good performance and to fulfil all the recommended conditions of SCCs.

Few investigations have been conducted on the microstructure of SCCs made with RA. Unlike studies on conventional concrete, which showed the performance of recycled aggregate concrete is mainly due to the porosity caused by the old paste in RA [10,11,12], this porosity leads to a weak interfacial zone between the new paste and the old one [13]. Hence, this low bond is attributed to both porosity and to the high absorption capacity of the RA [14]. It is clear, therefore, that the microstructure of the recycled aggregate concrete (RAC) is different from conventional one, since RA contain a proportion of old cement paste. In order to understand the behavior of RAC, it is necessary to study its porosity and its pore distribution, not only in the interface of transition zone (ITZ) between an RA and the new paste, but also in the bulk new paste. The most widely used method to determine the pore structure is mercury intrusion porosimetry (MIP) [15,16,17,18,19]. However, MIP has limitations in measuring actual pore size distributions and detecting pore diameters below 3 nm and above 375 µm, because pores are too small or too isolated to be filled by mercury. Nonetheless, MIP still has a great capacity to estimate total porosity and a characteristic pore size [19]. Some authors have investigated the microstructure of RAC with this technique [20,21,22,23,24,25]. Results show that the substitution of natural aggregates (NA) with RA increased porosity. Shicong et al. [20] showed that RA modify tailings pore size distribution with higher intrusion volumes in pores which diameters larger than 0.01 µm in RAC compared to natural aggregate concrete (NAC). The conclusion was that the porous old paste adhered to RA. Similar findings were observed by Uchikawa et al. [21]. Previous literature [22,23,24,25] also indicated that the replacement of RA correlates with the total volume and pore size. In addition, it demonstrated that the influence of RA was more important at lower ages, and that its effect was reduced while increasing the curing duration. Major quantitative changes were observed with the increase in pore volume for those with a radius below 30 nm. Gonzalez-Corominas et al. [24] used MIP to investigate the influence of steam curing on the pore structure of concrete containing different qualities of RA. Their results showed the effectiveness of steam curing in the refinement of pore structure. Furthermore, although the lowest quality RA had a coarser pore size distribution, the highest reductions—especially with respect to the capillary pores (10 µm–0.01 µm)—were observed in this material. This observation is explained by the internal curing effect of porous aggregates that enlarges binder hydration. The use of silica fume [25] caused a 32% reduction in the cement paste porosity with respect to reference concrete. However, the representational value of the sample for the MIP test in the case of (RAC) was not taken into account [20,21,22,23,24,25].

The different components of an RAC (RA, new paste and NA) each generally occupy a significant volume, in the range of tens of cubic centimeters. On the other hand, MIP does need a small volume of the sample for testing (a few cm^3^), raising the question of RAC sample representativity of a RAC sample with respect to the real mix. Therefore, the current porosity will be fully identified using other techniques. Hence, it is necessary to propose a new approach to study the microstructure of RAC by MIP test. A suitable sampling method, especially considering the lower volume of coarse aggregate in SCC compared to conventional concrete, might ease the sampling of the new paste. Accordingly, this work proposes a sampling method to study the microstructure of the new paste (NP) of self-compacting concrete (SCC) for different levels of recycled aggregates (RA). The binder content was kept constant for all concrete mixtures, with a water to binder ratio (w/b) of 0.5. SCC mixtures were prepared with 0%, 30%, 50% and 100% of coarse RA. The pore distribution of the new paste was evaluated by MIP test, and its anhydrous content was quantified by SEM images analysis. Additionally, the contribution of the porosity of NP and RA to the decrease in compressive strength was investigated and demonstrated.

## 2. Materials and Methods

### 2.1. Materials

The type of cement used in all mixtures was CEM I/42.5 N with a real density of 3.16 g/cm^3^ and a blaine specific surface of 3150 g/cm^2^. A limestone filler (LF) was used with a real density of 2.70 g/cm^3^ and a blaine specific surface of 4200 g/cm^2^. The chemical composition of both materials is provided in Table 1. A superplasticizer (SIKA VISCOCRETE TEMPO 12) enhanced the workability of SCC.

**Figure 1:**
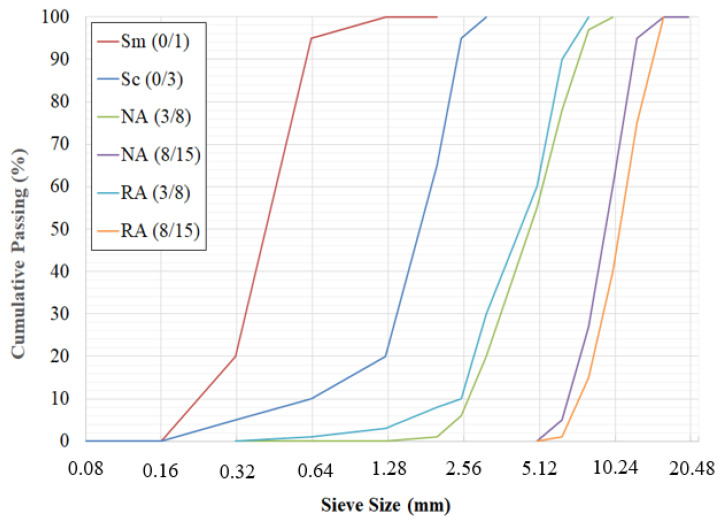
Grading curves of different types of aggregates.

To obtain an SCC, the volume of the aggregates and their maximum size has been reduced to mitigate friction and to avoid blockages in confined areas. The maximum diameter of the coarse aggregates was fixed in our study at 15 mm. In this study, siliceous sand and crushed limestone sand were used as the fine natural aggregates. Natural coarse aggregates were made of crushed limestone aggregates and recycled coarse aggregates were obtained from construction and demolition waste (CDW). The grading curves of aggregates are presented in Figure 1.

The main properties of the aggregates are displayed in Table 2, where Dr is the relative density of the particles, A is the water absorption, and LA is the Los Angeles fragmentation coefficient. These properties were measured according to the NF-EN-1097-6 standard [26]. The porosity P was determined according to the NF P 18-459 standard [27,28].

### 2.2. RA Pre-Saturation Process

In order to achieve equal consistency, and due to the higher water rate absorption of RA, the mixing of water had to be adjusted. This adjustment was achieved by using RA under the saturated surface dry (SSD) condition. If the aggregates are fully saturated, the surface moisture would increase the effective w/c ratio, the aim being that RA would have the same water absorption coefficient as NA. Thus, for each fraction of RA (3/8) and (8/15), the amount of water pre-saturation (M W.pre-sat) is defined as the difference between the water absorbed by RA and NA, and is calculated as follows:

% Wpre-sat = % abs (RA) − % abs (NA)(1)

M W.pre-sat = % Wpre-sat x M(2)

With M the mass of RA during mixing

Despite the very rapid evolution of water absorption of the RA in the first five minutes, a pre-saturation time for 24 h was chosen. This is consistent with the relevant literature, in which it is indicated that absorption lasts for hours after its initial quick step [29,30]. This guaranteed that no water was absorbed after mixing, when pre-saturation is carried out for 5 min. Before each mix, the aggregates were placed in a large plastic bottle for 24 h and the pre-saturation water was added residually. Great care was taken when rolling the bottle to avoid any air entrance and to ensure all the aggregates were impregnated. This operation was conducted for a period of 30–45 min. The bottle method was also adopted in the case of recycled sand [31], and the aggregates were left in their dedicated flask.

### 2.3. Design of Concrete Mixes

The mixing method used to design the compositions of the SCC is based on a composition of one with limestone fillers. The amount of components required for making one cubic meter of concrete was constant. They consisted in a paste volume of 37.5% (375 L/m^3^), a water to binder ratio w/b ratio of 0.5 and a gravels/sand (G/S) ratio close to one. SCC-RA mixes were designed by replacing a volume of NA with RA. Four mixes were prepared with 0%, 30%, 50% and 100% recycled aggregates respectively labelled to SCC0, SCC30, SCC50 and SCC100 respectively. The mix designs of the concrete are shown in Table 3.

All SCC mixes showed a slump flow in the 700 mm-range and a good resistance to segregation. For each composition, cylindrical specimens of 16 × 32 cm were casted in steel molds, and cured in water at 20 ± 2 °C. The compressive strength of these concrete cylinders was measured on three samples according to NF EN 12390-3 [32] with a hydraulic press capacity of 2400 kN.

### 2.4. MIP Tests

Mercury intrusion porosimetry (MIP) is one of the main methods for investigating the mesoporous structure (pore radius between 2 and 50 nm) and the macro-porous nature (apertures greater than 50 nm) of cementitious materials. Its effectiveness is based on the principle that to fill a non-wetting fluid into a pore of diameter d, a pressure P inversely proportional to this diameter must be applied. This pressure is given by the Washburn Equation (3):

(3) $$P=\frac{-4\;\gamma cos\theta }{d}$$

Here, the surface tension γ of mercury is of 485 N/m, and the contact angle θ between mercury and the pore wall of 141.3°. MIP tests were conducted on an AutoPore IV 9500 V1.09, from Micrometrics Instrument Corporation, under a maximum pressure of 413 MPa to reach pores with a radius of 3.6 nm.

**Figure 2:**
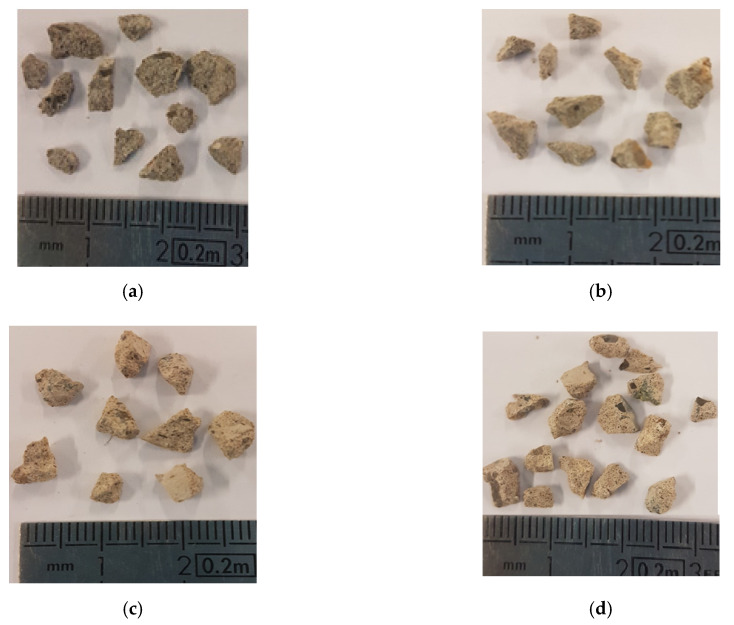
Samples of new pastes before MIP tests (**a**) NP0, (**b**) NP30, (**c**) NP50 and (**d**) NP100.

RA concretes (SCC30 and SCC50) are considered as multiphase composites in which the new paste (NP) is joined into coarse NA and coarse RA. The new paste consists of the binder and the sand, and represents 69% v/v of concrete. SCC0 consists in NP and coarse NA, while SCC100 in NP and coarse RA. As the common phase between the different concretes is the new paste, the study and the comparison between mixes focused on its microstructure. Each concrete type was sampled (approximate volume 2 cm^3^), as shown in Figure 2, and extracted from 16 × 5 cm discs. The tested NP samples extracted from SCC0, SCC30, SCC50 and SCC100 were respectively labelled NP0, NP30, NP50 and NP100.

These samples were first introduced into the liquid nitrogen for 5 min in order to stop the hydration. They were then lyophilized in an alpha freeze-dryer 1–4 Ld for 48 h to extract the liquid water by sublimation. The samples were stored in a desiccator until testing. This technique has proven to be the most appropriate one to limit microstructural damage [33].

### 2.5. SEM Observations

**Figure 3:**
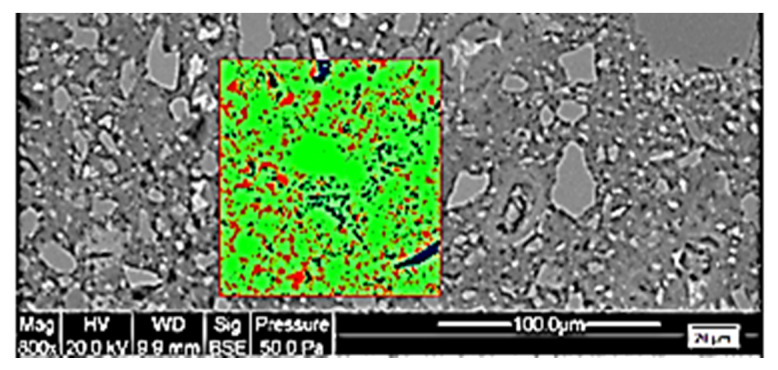
Example of SEM image analysis with a magnification of 800 on a NP0 sample.

The study focused on the anhydrous content for the new paste (NP0, NP30, NP50 and NP100) of each concrete type. The study of the concrete microstructure of all RA-SCC samples was performed using a scanning electron microscope (SEM), Quanta 400 from FEI Company. Their observation was carried out through electron backscattered imaging (BSE) to identify the different phases of the microstructure. Before initiating SEM observation, to remove the free water, small samples of size 35 × 35 × 10 mm were dried under vacuum with silica gel at 45 °C for 14 days. The dry samples were then impregnated with epoxy resin; then, the samples were polished in various steps to create a smooth plane surface for SEM imaging. Because our samples were not conductive, we had to add a very thin gold metallic coat. Obtained BSE images were processed with the Stream Motion software to distinguish the different phases of the samples using a toll dedicated to contrast the grey levels. Images of NP were taken at random spots, with an analysis over a 10,000 µm^2−^ square on images with a magnification of 800 times, as illustrated in Figure 3. As shown in a previous study [34], the porosity appears red, the anhydrous are blue, and the hydrated cement gel appears green (see Figure 3). It took approximately 30 images to cover new paste on three samples of each type of concrete.

## 3. Results

### 3.1. Compression Strengths

**Figure 4:**
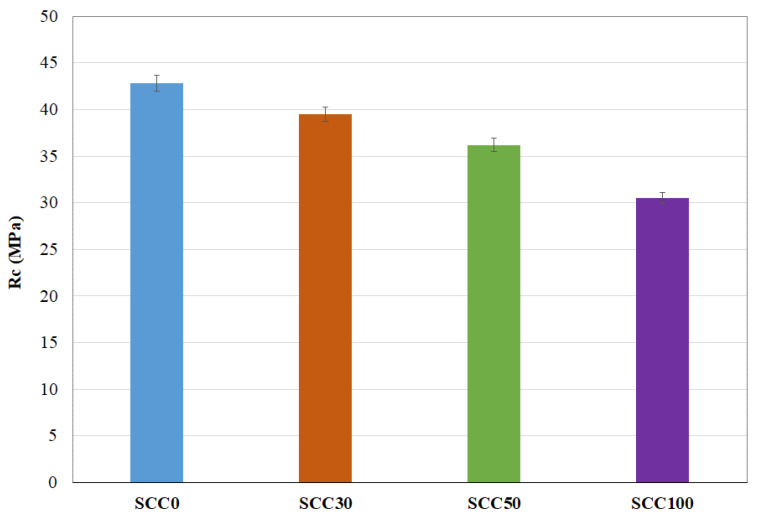
Compressive strengths results at 90 days.

Figure 4 shows the compressive strengths of SCC as a function of their RA content. The results obtained have good repeatability with a standard deviation almost constant. A 90-day compressive strength decrease can be seen with the increase in the RA content.

This result is widely accepted and consistent with the literature [8,35]. Some authors attribute this compressive strength decrease to a high total porosity of RCA concrete [22], while others to the high porosity in the ITZ [36,37]. Strengths losses are then linked to the presence of old paste, without taking into account the modifications of the NP microstructure. The presence of the old paste leads to high porosity and, consequently, the compressive strength decreases. However, and as will be demonstrated later in this study, the microstructure of the NP is affected by the presence of RA once a replacement threshold is reached. This is why a specific study of the NP microstructure based on the MIP test was undertaken.

### 3.2. Influence of Recycled Aggregates (RA) on the Porous Structure of the New Paste (NP)

**Figure 5:**
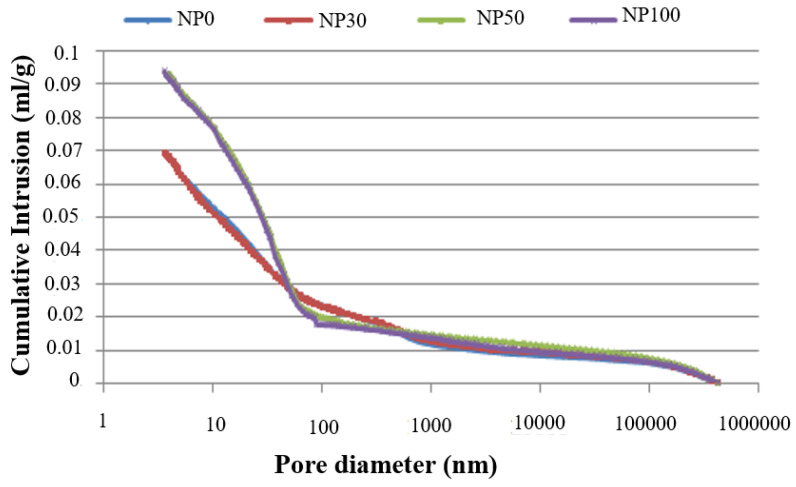
Cumulative intruded pore volume of new pastes (NP).

Figure 5 shows the cumulative intrusion volume as a function of pore diameter for various the tested NP. The greater the RA percentage, the larger the total mercury intrusion. Two groups of pore size distributions can be distinguished. The first one for NP0 and NP30 evolves similarly with a total introduced pore volume of 0.069 mL/g, while a second one for NP50 and N100 pastes present a total introduced pore volume of 0.09 mL/g. In both groups, a slope change is observed for pore diameter of 100 nm.

**Figure 6:**
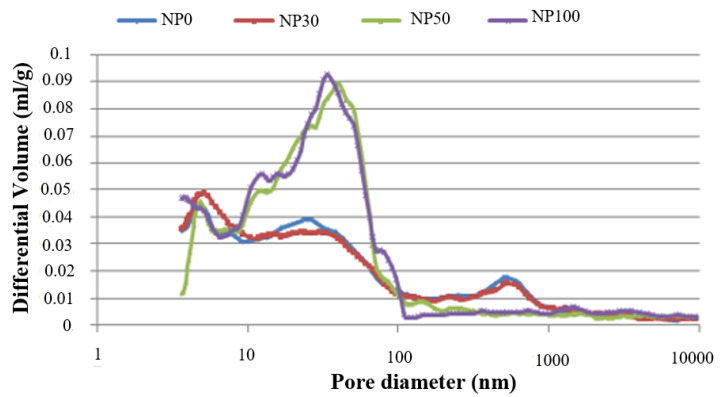
Pore size distribution for the different new pastes (NP).

Differential intrusion curves of the NP in Figure 6 provide extended information on pore structure. Generally, in cured cement paste, two different pore diameters are considered. The pore diameter corresponding to the first peak occurs when the mercury intrusion is through a porous network connected to the surface of the sample. It is defined as the critical pore diameter (Dc) of the capillary pores that varies from 10 nm to 10,000 nm [38]. The second peak is related to gel pores, and according to study [39], its value is less than 10 nm, while some other authors are providing a value in the 20–40 nm range [40].

In the present study, NP0 and NP30 have a pore diameter distribution spread all over the whole measured range. No clear visible peaks in the capillary pores were observed besides the ones at 5 and 4.35 nm. A less rounded peak at the capillary pores for NP0 and NP30 is attributed to a large pore size distribution for this material phase, as stated by [38]. It is possible that these peaks correspond to an intrusion in the material phase with a distinct network of smaller pores. A less pronounced peak in these pastes, therefore, corresponded to a more or less connected pore path.

For NP50 and NP100 pastes, a sharp peak is visible around 50–60 nm. It is associated with the minimum diameter of an interconnected capillary network, called the critical diameter. This diameter is determined from the inflection point of the curves in Figure 5, and summarized in Table 4. Other peaks could also be observed for the range of mesopores, at 11 nm for the NP100, and at 4.60 nm for both NP50 and NP100 related to gel pores.

**Figure 7:**
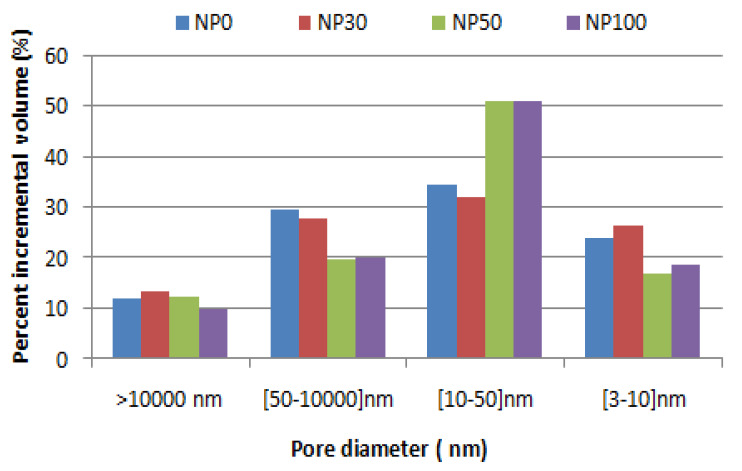
The pore percentage of new pastes (NP) for different pore diameter ranges.

Figure 7 shows the total mercury intrusion pore volume for several pore size ranges. NP50 and NP100 pastes, despite their high total porosity, have a finer pore distribution than NP0 and NP30 pastes in the pore aperture below 50 nm. By means of the wet RA, a water movement between the new paste and the RA is created, which disturbs the hydration of the new paste and initiates two hypotheses. In the first, moisture contained in the pores of the paste is gradually released to allow a continuous hydration [41,42], which leads to the refinement of the pore structure [24]. In the second, RA absorbs mixing water, reducing the w/c ratio in the NP. This observation made it necessary to implement further investigations. For this, SEM observations were carried out to examine the microstructure of the new paste.

### 3.3. Influence of Recycled Aggregates (RA) on the Anhydrous Content of the New Paste (NP)

**Figure 8:**
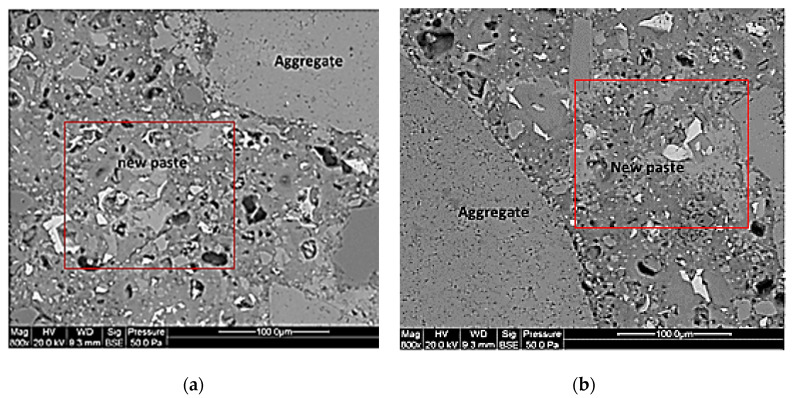

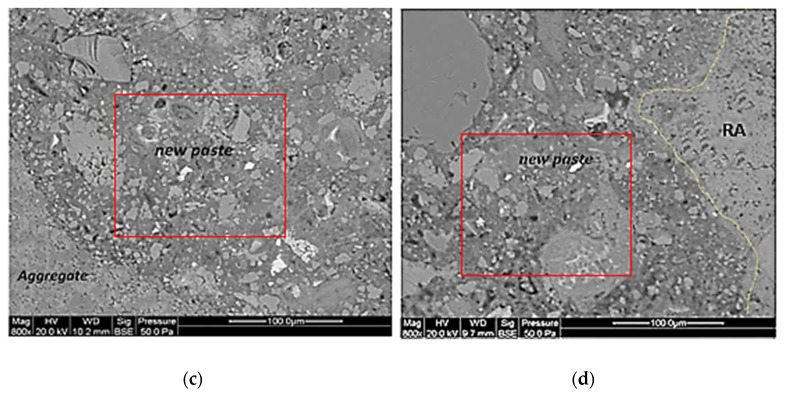
SEM images of new pastes (**a**) NP0, (**b**) NP30, (**c**) NP50 and (**d**) NP100.

The SEM observations conducted on the NP of each SCC enables us to evaluate anhydrous content. The content of the anhydrous grains (white elements in Figure 8) in the new paste change from one concrete to the next. Image analysis evaluations show that the anhydrous content in the new paste decreases with an increasing RA replacement percentage. The anhydrous content was 5.75% for NP0 and 5.04% for NP30, while it was 3.63 % for NP50 and 1.21% for NP100. This validates the first hypothesis, which is that the water absorbed by the RA during the pre-saturation process can migrate into the new paste, which generates less anhydrous and macrospores for high RA-substitution rate. Cement hydration continues due to the availability of additional water, as has already been observed by Djerbi [29].

Figure 7 shows that the percentage of macrospores of sizes between 50 and 10,000 nm is nearly 30% for both NP0 and NP30, respectively, and only 20% for NP50 and NP100. This phenomenon has made it possible to generate a finer pore distribution in the existing mesopore network. It can be seen that the percentage of pores in the 10–50 nm range is of the order of 51% for NP50 and NP100, compared to only 34% and 32%, respectively, for NP0 and NP30. Within the 3–10 nm pore radius range, the NP0 and NP30 develop higher gel pore rates than the other two pastes. This explains the convergence of the peaks towards the areas of the small pores and the disappearance of that at the level of the capillary pores. Therefore, as the hydration moves forward, the hydrates formed fill the pores and lead to the movement of the first peak towards a finer pore diameter [43]. The comparison between the porosity of RA and NP from Table 4 shows that RA has a total porosity 15% greater than that of NP0 and NP30. For high substitution rates, it shows a decrease of about 9% for NP50 and NP100.

### 3.4. Contribution of the Porosity of NP and RA to the Decrease in Compressive Strength

**Figure 9:**
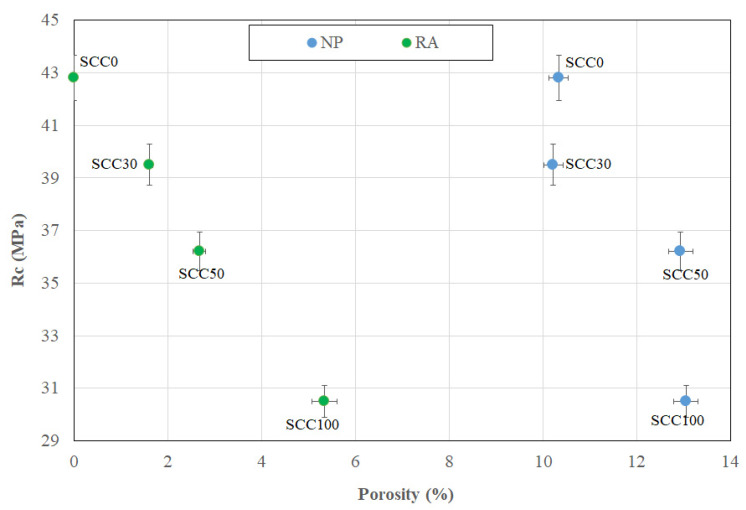
Relationship between porosity of RAC component and compressive strength.

In order to study the effect of the porosity of each component of concrete, it is important to relate the porosity to the volume of each component present in the concrete. The volume of the coarse aggregates (>3 mm) is 31% in the total volume of concrete (see Table 3). An estimate of the porosity of the coarse RA evaluated by MIP test for each substitution rate is 30% for RA30-SCC30, 50% for RA50-SCC50, and 100% for RA100-SCC100. The volume of the NP is 69%, also taking into account the sand fraction (see Table 3). Figure 9 shows the contribution of the porosity of RA and that of NP for each concrete type in the decrease in compressive strength (Rc). The reduction in compressive strength between SCC0 and SCC30 can be attributed to RA porosity. While the reduction in the compressive strength between SCC30 and SCC50 is due to NP porosity increase and RA porosity of about 26% and 66%, respectively; this increase in porosity causes a decrease in Rc of about 8%. On the other hand, the decrease in Rc of 16% between SCC50 and SCC100 is only generated by the increase in RA porosity by a factor of 2.

## 4. Conclusions

The modification of the microstructure of the new paste (NP) of self-compacting concrete (SCC) for different levels of recycled aggregate (RA) at 90 days compared that to the reference concrete has been explored. The results obtained from this work can be summarized as follows:

- The microstructural characterization of self compacting concretes (SCC) containing recycled aggregates (RA) with the proposed sampling method enables us to evaluate the pore network of their different components as RA and NP.
- The replacement of coarse aggregates by RA at 30% does not affect the new paste of SCC. The pore size distribution of NP30 is similar to the reference concrete NP0 with a total introduced pore volume of 0.069 mL/g. While NP50 and N100 pastes present a total introduced pore volume of 0.09 mL/g.
- The porosity of the new paste presents a significant variation for 50% and 100% replacement in RA with respect to NP0 and NP30. NP50 and NP100 exhibit higher refinement of the porous structure. The number of macrospores of a diameter between 50 and 10,000 nm is reduced by 20% for NP50 and NP100 in comparison to NP0 and NP30, of which the reduction is about 30%. This is attributed to the release of water absorbed by the RA during the pre-saturation process that allows continuous hydration.
- The anhydrous rates in the NP decrease with increasing RA replacement percentage. The anhydrous content was 5.75% for NP0 and 5.04% for NP30, while it was 3.63% for NP50 and 1.21% for NP100. This validates the hypothesis that the water absorbed by RA during the pre-saturation process can migrate into the NP, generating less anhydrous and macrospores for high rate substitution of RA.
- The compressive strength loss for RA concretes SSC50 and SCC100 is influenced not only by the porous old paste of RA, but also by the porosity of the new paste. The paste is similar for these two concretes and shows an increase of 26% compared to the reference concrete.
- Further works will be conducted to study the microstructure evolution between 28 days and 90 days.

## Figures and Tables

**Table 1:** Chemical composition of cement and limestone filler (LF).

Compound (%)	SiO_2_	CaO	MgO	Fe_2_O_3_	Al_2_O_3_	SO_3_	K_2_O	Na_2_O	LOI
Cement	21.8	64.7	1.62	6.37	3.88	0.42	0.33	0.22	0.66
LF	5.19	49.35	1.29	1.29	1.50	0.06	0.27	0.12	40.93

**Table 2:** Properties of the aggregates.

Aggregate Size (mm)	Dr (g/cm^3^)	A (%)	P (%)	LA (%)
Siliceous sand Sm (0/1)	2.60	1	-	-
Crushed limestone sand Sc (0/3)	2.68	1.8	-	-
Natural coarse aggregates NA (3/8)	2.66	1.4	-	-
Natural coarse aggregates NA (8/15)	2.64	1.3	-	21
Recycled coarse aggregates RA (3/8)	2.35	6.1	18.7	-
Recycled coarse aggregates RA (8/15)	2.42	4.8	16.4	33

**Table 3:** Mix composition of the concretes, per cubic meter.

Mixture(kg/m^3^)	SCC0	SCC30	SCC50	SCC100
Cement	382	382	382	382
LF	65	65	65	65
Sm (0/1)	578	578	578	578
Sc (0/3)	253	253	253	253
NA (3/8)	335	235	168	0
NA (8/15)	495	347	248	0
RA (3/8)	0	89	147	296
RA (8/15)	0	136	227	454
Effective water	224	224	224	224
w/b (C + LF)	0.5	0.5	0.5	0.5
Superplasticizer (%)	2.67	2.67	2.67	2.67

**Table 4:** Porosity, total intruded mercury volume and critical pore size of NP and RA.

	Porosity (%)	Mercury Volume (mL/g)	Dc(nm)
NP0	14.99	0.0693	-
NP30	14.80	0.0691	-
NP50	18.75	0.093	40.24
NP100	18.92	0.094	33.80
RA	17.26	0.084	21.19
